# Prokineticin 2 antagonist, PKRA7 suppresses arthritis in mice with collagen-induced arthritis

**DOI:** 10.1186/s12891-016-1243-0

**Published:** 2016-09-08

**Authors:** Haruyasu Ito, Kentaro Noda, Ken Yoshida, Kazuhiro Otani, Masayuki Yoshiga, Yohsuke Oto, Saburo Saito, Daitaro Kurosaka

**Affiliations:** 1 Division of Rheumatology, Department of Internal Medicine, The Jikei University School of Medicine, 3-25-8 Nishi-shimbashi, Minato-ku, Tokyo Japan; 2 Division of Molecular Immunology, Research Center for Medical Sciences, The Jikei University School of Medicine, 3-25-8 Nishi-shimbashi, Minato-ku, Tokyo Japan

**Keywords:** Prokineticin 2, Prokineticin receptor 1, Prokineticin receptor 2, Prokineticin antagonist, PKRA7, Collagen-induced arthritis

## Abstract

**Background:**

Prokineticin 2 (PK2) expression is upregulated in mice with collagen-induced arthritis (CIA), an animal model of rheumatoid arthritis. The purpose of our study was to investigate the effects of PK2 inhibition on CIA.

**Methods:**

PK2, prokineticin receptor (PKR) 1, and PKR2 mRNA transcripts in the joints of CIA mice were measured by real-time PCR on Days 21, 28, and 35 (*n* = 15/day). Localization of PKR1 and PKR2 proteins was examined immunohistochemically. PKRA7, a PK2 antagonist, was administered intraperitoneally for 2 weeks to CIA mice, and the severity of arthritis was compared between treated (*n* = 12) and untreated (*n* = 12) mice. The gene expression levels of inflammatory cytokines IL-1β, IL-6, TNF-α, and VEGF were also measured by real-time PCR and compared between treated (*n* = 6) and untreated (*n* = 6) CIA mice. The data was statistically analyzed, and *P* values of less than 0.05 were considered significant.

**Results:**

In the thickened synovial membrane, PKR1 protein was expressed in infiltrating neutrophils, while PKR2 expression was found in macrophage-like mononuclear cells. PK2 gene expression was significantly more pronounced on Days 28 and 35 than on Day 21 (2.15 and 2.03 versus 1.00, *P* = 0.0311 and 0.0247; Dunn’s multiple comparison). PKR2 gene expression levels were significantly higher on Days 28 and 35 compared to Day 21 (25.4 and 39.3 versus 1.0, *P* = 0.002 and < 0.0001; Dunn’s multiple comparison). Administration of PKRA7 suppressed the severity of arthritis (*P* < 0.001; two-way analysis of variance). A gene expression analysis of inflammatory cytokines revealed significantly reduced IL-1β and lL-6 expression in the joints of PKRA7-treated mice compared to untreated mice (0.1 versus 1.0, *P* = 0.0043 and 0.04 versus 1.0, *P* = 0.0022, respectively; Mann-Whitney test).

**Conclusions:**

PK2 inhibition suppressed arthritis in mice with CIA.

## Background

Prokineticin 2 (PK2) is a large-class secreted peptide containing a five-disulfide-bridged motif called a colipase fold [1]. Two types of G-protein-coupled receptors, prokineticin receptor 1 (PKR1) and prokineticin receptor 2 (PKR2), have been identified as the target of PK2. PK2 and its receptors are expressed in various tissues, e.g., the testis, skin, lung, intestine, ovary and the central nervous system as well as circulating neutrophils, macrophages and lymphocytes [2–5], and involved in diverse physiological functions such as angiogenesis, neurogenesis, circadian rhythm, and pain thresholds [1, 6–9]. Abnormalities in PK2-PKR1/2 signaling have been associated with various human diseases, including polycystic ovarian syndrome, myocardial infarction, colorectal cancer, and Kallmann syndrome [10–12]. In light of the fact that angiogenesis plays an important role in the pathogenesis of rheumatoid arthritis [13, 14], we previously investigated PK2 expression in mice with collagen-induced arthritis (CIA), the animal model of rheumatoid arthritis, and reported that PK2 gene expression was significantly elevated in the joints of CIA mice and correlated with the severity of the arthritis [15]. More recently, such upregulation of PK2 has been reported also in a rat testitis model [16], a mouse model of inflammatory colitis [17], and a mouse model with autoimmune encephalomyelitis [18]. In in vitro studies, PK2 has been shown to induce proinflammatory cytokine production by macrophages and splenocytes [2, 3]. Taken together, these findings point to PK2’s role in various inflammatory diseases.

**Fig. 1:**
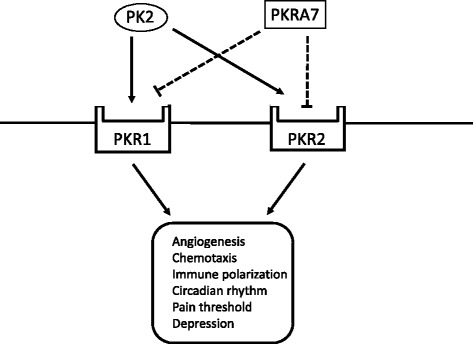
PKRA7 as an antagonist of PK2. PK2 binds to PKR1 and PKR2 receptors and regulates various physiological processes, including angiogenesis, chemotaxis, immune polarization, and circadian rhythm. PKRA7 also binds to PKR1 and PKR2 and competitively blocks the binding of PK2

PKRA7 is a small molecular inhibitor developed by Curtis et al. by substitution of alanine with methionine and addition of methionine at the N-terminus of PK2; the mutated peptide mimics PK2 and competitively blocks PK2-PKR1/2 interactions [19] (Fig. 1). The researchers showed that this molecule inhibits angiogenesis and macrophage infiltration in mice transplanted with glioblastoma and pancreatic cancer, respectively [19]. The primary goal of our study was to investigate the effects of PKRA7 on arthritis using the mouse model with CIA.

## Methods

### CIA mice

All animal experiments were approved by the Institutional Animal Care and Use Committee of the Jikei University, Tokyo, Japan. Five-week-old DBA/1 J mice (*n* = 69) were purchased from Sankyo Labo Service (Tokyo, Japan) and immunized intradermally at the dorsal root of the tail with bovine type II collagen (200 μg/mouse; Collagen Research Center, Tokyo, Japan) emulsified in Freund’s complete adjuvant (Becton Dickinson and Company, NJ, USA) (Day 0). On Day 21, a booster injection of bovine type II collagen emulsified in Freund’s incomplete adjuvant (Becton Dickinson and Company) was administered in the same manner. The severity of arthritis was evaluated by a clinical rheumatologist (study co-author KO), who was blinded to the study groups, and expressed as the sum of the scores for all four limbs as assessed on the following scale: 0, normal; 1, swelling of digits alone or mild swelling of wrist and ankle joints; 2, clear swelling of wrist and ankle joints; and 3, severe swelling of wrist and ankle joints.

### Gene expression analysis of joints

CIA mice were sacrificed under isoflurane anesthesia. The forelimbs and hindlimbs were amputated 5 mm proximal to the wrist and ankle joints, and RNA was extracted using an RNeasy Lipid Tissue Mini Kit (Qiagen, Tokyo, Japan). Real-time PCR was performed on an Applied Biosystems StepOne Plus Real-Time PCR System (Thermo Fisher Scientific, MA, USA) using TaqMan probes and primers (Thermo Fisher Scientific) for PK2 (Mm00450080_m1), PKR1 (Mm01204733_m1), PKR2 (Mm00769571_m1), IL-1 (Mm01336189_m1), IL-6 (Mm00446190_m1), TNF-α (Mm00443258_m1), VEGF (Mm01281449_m1), and β-actin (Mm00607939_s1). Gene expression levels were analyzed by the ΔΔCT method using β-actin as a reference gene.

The gene expression levels of PK2, PKR1, and PKR2 were analyzed for the CIA mice sacrificed on Days 21, 28, and 35. The gene expression levels on Days 28 and 35 were expressed relative to the arbitrary assigned value on Day 21 (=1.0). The experiment was repeated 3 times for each group of five mice (5 mice × 3 days × 3 repetitions = 45 mice). The correlation between arthritis scores and PK2, PKR1, and PKR2 gene expression levels was analyzed for all 45 mice. Separately, cytokine gene expression levels were analyzed for the PKRA7-treated and -untreated mice (*n* = 6 each) sacrificed on Day 35 and were expressed relative to the values obtained for untreated mice.

### PKR1 and PKR2 protein analysis

For immunohistochemical staining of PKR1, isolated joints on Day 35 were perfusion-fixed, decalcified, and embedded in optimal cutting temperature compound (Sakura Finetek, CA, USA) for frozen sectioning (at a thickness of 7 μm). For PKR2 immunostaining, the tissues were embedded in optimal cutting temperature compound without fixation or decalcification and cryosectioned. Endogenous peroxidase was inactivated with a peroxidase blocking reagent (Dako, Glostrup, Denmark). Sections were incubated with a primary antibody, either rabbit anti-mouse PKR1 antibody (Covalab, Villeurbanne, France) or rabbit anti-mouse PKR2 antibody (Covalab), and then with a secondary antibody (Simple Stain Rabbit Max PO; Nichirei Bioscience, Tokyo, Japan). Simple Stain DAB Solution (Nichirei Bioscience) was used for visualization of the secondary antibody. The sections were then counterstained with hematoxylin and examined under a light microscope (Axio Imager A1, Carl Zeiss, Göttingen, Germany).

### Administration of PKRA7

DMSO was used as a vehicle. Prior to administration, PKRA7 in DMSO was diluted with PBS to adjust the concentration of DMSO to 5 %. CIA mice received either 15 mg/kg/day of PKRA7 or 5 % DMSO (control) for 2 weeks from Day 21. The experiment was repeated twice in groups of 6 mice (*n* = 12). On Day 35, the mice were sacrificed and their limbs amputated. Isolated joints were fixed, decalcified, and embedded in paraffin, and the blocks were sectioned into slices at a thickness of 4 μm. Sections were stained with hematoxylin and eosin and examined under an Axio Imager A1 microscope. The gene expression levels of inflammatory cytokines IL-1β, IL-6, TNF-α, and VEGF were also measured by real-time PCR and compared between PKRA7-treated (*n* = 6) and -untreated (*n* = 6) CIA mice on Day 35.

### Statistical analysis

The gene expression levels of PK2, PKR1, and PKR2 on Days 21, 28, and 35 were analyzed by a Kruskal-Wallis test with a Dunn’s multiple comparison test *post hoc*. Spearman’s rank correlation coefficients were used to examine the correlation between arthritis scores and relative gene expression. Arthritis scores for untreated and treated mice were compared by two-way analysis of variance with a Sidak’s multiple comparison test *post hoc*. Cytokine gene expression was analyzed by a Mann-Whitney test. All results were expressed as mean values ± SEM.

## Results

**Fig. 2:**
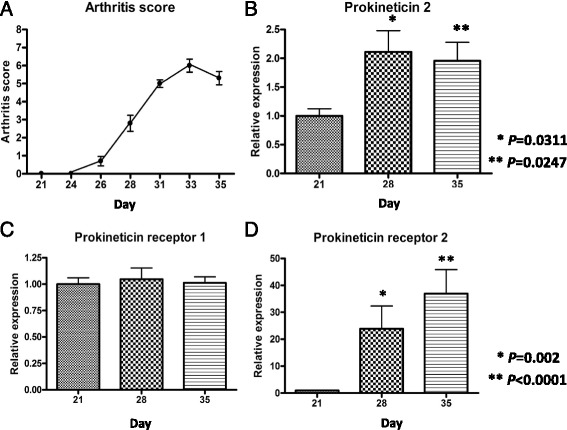
Changes in arthritis scores and PK2, PKR1, and PKR2 gene expression before and after the onset of arthritis. Arthritis was experimentally induced with a collagen injection on Day 21. **a** The severity of arthritis peaked on Day 33 and decreased thereafter. **b** PK2 gene expression in the joints was more elevated on Days 28 and 35 than on Day 21. **c** There was no significant change in PKR1 gene expression levels before and after the onset of arthritis. **d** PKR2 gene expression was significantly more pronounced on Days 28 and 35 than on Day 21. Values are relative to the arbitrarily assigned value of 1.0 for Day 21 and are given as means ± SEM. *P* values by Dunn’s multiple comparison

**Fig. 3:**
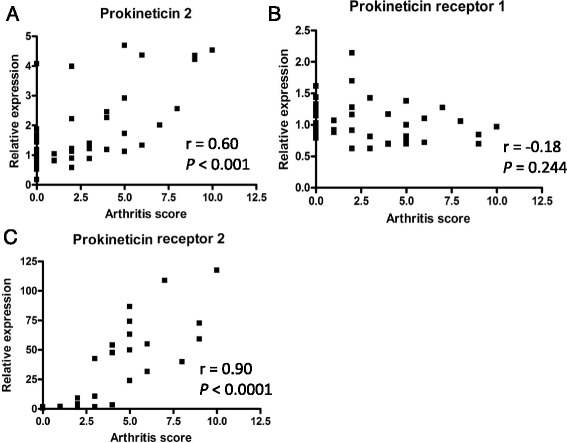
Correlation between arthritis scores and PK2, PKR1, and PKR2 gene expression. Severity scores were significantly correlated with **a** PK2 and **c** PKR2 gene expression levels but not with **b** PKR1 gene expression based on Spearman’s rank correlation coefficients (r). Data was obtained from mice sacrificed on Days 21, 28, and 35

Changes in arthritis scores in CIA mice are shown in Fig. 2a. Scores peaked on Day 33 and decreased thereafter. Real-time PCR results showed that PK2 gene expression was significantly more pronounced on Days 28 and 35 than on Day 21 (Fig. 2b; 2.15 and 2.03 versus 1.00, *P* = 0.0311 and *P* = 0.0247, respectively). PKR1 gene expression levels were similar on all days (Fig. 2c), while PKR2 gene expression levels were significantly higher on Days 28 and 35 compared to Day 21 (Fig. 2d; 25.4 and 39.3 versus 1.0, *P* = 0.002 and *P* ≤ 0.0001, respectively). The Spearman’s rank correlation test revealed a significant correlation between arthritis scores and relative PK2 gene expression levels with a correlation coefficient of 0.60 (Fig. 3a, *P* < 0.001). Arthritis scores were not correlated with PKR1 gene expression levels (Fig. 3b) but were significantly correlated with PKR2 gene expression levels (Fig. 3c, *P* < 0.0001).

**Fig. 4:**
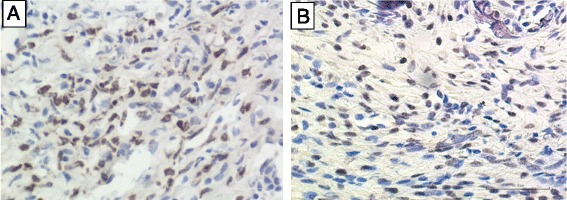
Immunostaining of PKR1 and PKR2 proteins in synovial tissue. Brown staining indicates PKR1- or PKR2-positive cells. **a** Neutrophils infiltrating in the synovium membrane of CIA mice were positive for PKR1 (400×). **b** PKR2-positive cells were macrophage-like cells (400×)

Immunohistochemical staining of PKR1 and PKR2 proteins showed that PKR1-positive cells were predominantly neutrophils infiltrating in the synovial membrane (Fig. 4a). PKR2-positive cells were also found in the synovium but associated with macrophage-like mononuclear cells (Fig. 4b).

**Fig. 5:**
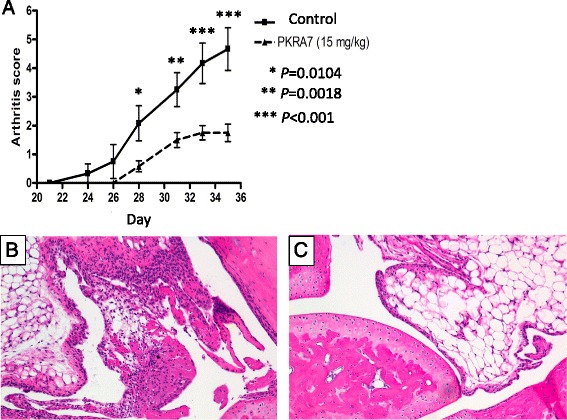
Effects of the PK2 antagonist PKRA7. **a** Arthritis scores were significantly lower in PKRA7-treated mice compared to control mice. *P* values by Sidak’s multiple comparison. **b** Hematoxylin and eosin staining of the ankle joint synovial membrane of control CIA mice (100×) on Day 35. **c** Hematoxylin and eosin staining of the ankle joint synovial membrane of PKRA7-treated CIA mice (100×) on Day 35. Note milder inflammatory cell infiltration and synovial thickening

**Fig. 6:**
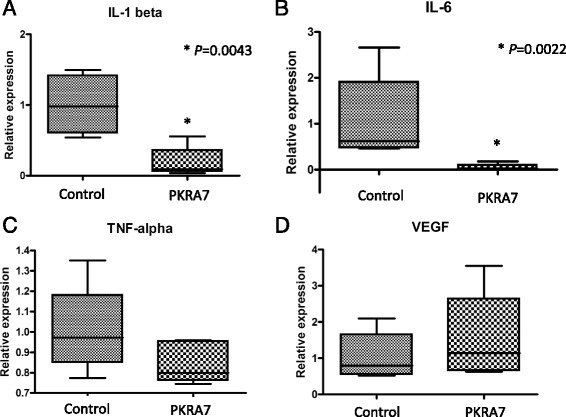
Comparison of cytokine gene expression in the joints of PKRA7-treated and untreated CIA mice on Day 35. **a** IL-1 gene expression was significantly less pronounced in PKRA7-treated mice (*P* = 0.0022). **b** IL-6 gene expression was also significantly reduced by PKRA7 (*P* = 0.0043). **c** TNF-α levels were numerically lower for PKRA7-treated mice but did not differ in a statistically significant manner from values for control mice. **d** VEGF gene expression was similar without regard to PKRA7 treatment. *P* values by Mann-Whitney test

Daily intraperitoneal administration of the PK2 antagonist PKRA7 to CIA mice before the onset of arthritis from Day 21 resulted in significantly lower arthritis scores in treated mice compared to control CIA mice on Days 28, 31, 33, and 35 (Fig. 5a; *P* = 0.0104, *P* = 0.0018, *P* < 0.001 and *P* < 0.001, respectively). The mean arthritis score on Day 35 was 4.67 for the control group versus 1.75 for the PKRA7-treated mice. Histological evaluation of the synovium of the ankle joints showed less extensive inflammatory cell infiltration and synovial thickening in treated mice than in control mice on Day 35 (Fig. 5b, c). The gene expression analysis of inflammatory cytokine on Day 35 revealed significantly lower IL-1β and 1 L-6 expression levels in the joints of PKRA7-treated mice compared to control mice (Fig. 6a, b; *P* = 0.0043 and *P* = 0.0022, respectively). TNF-α gene expression levels were also lower in PKRA7-treated mice, albeit without statistical significance (Fig. 6c). There was no significant difference in VEGF gene expression between the two groups (Fig. 6d).

## Discussion

In the present study, we demonstrated that PKR1 protein was expressed in infiltrating neutrophils, while PKR2 protein was present in macrophage-like mononuclear cells in the synovial membrane of CIA mice. We also found that PK2 and PKR2 gene expression levels were elevated in the CIA joints and that administration of PKRA7 suppressed the severity of arthritis.

PKR1 is expressed in neutrophils, macrophages, and lymphocytes [2, 3, 5]. In inflamed joints, therefore, PKR1 expression is likely found in those blood cells infiltrating the synovial membrane. To demonstrate this fact, we performed immunohistochemical staining of PKR1 protein using a well-established mouse model of rheumatoid arthritis. We found that PKR1 was expressed in neutrophils in the synovial membrane. Although there was no increase in PKR1 gene expression, the gene expression levels of its ligand PK2 were elevated, indicating that PK2-PKR1 signaling was likely enhanced in those synovial neutrophils. In the light of the diverse roles of neutrophils in the development of rheumatoid arthritis [20], these results are encouraging for future studies focusing on the exact mechanism of the PK2-PKR1 pathway in the pathogenesis of arthritis in this model.

Like PKR1, PKR2 is expressed in neutrophils, macrophages, and lymphocytes in the blood [2, 3, 5]. We found that PKR2 protein was present in macrophage-like cells in the synovial membrane of CIA mice and that, unlike PKR1, PKR2 gene expression was more pronounced in inflamed joints. Furthermore, the level of PKR2 gene expression was significantly correlated with the severity of the arthritis.

To investigate the effects of PK2 inhibition on arthritis, we used the PK2 antagonist PKRA7. PKRA7 has been shown to decrease tumor size by inhibiting angiogenesis and macrophage infiltration when administered to mouse models of glioblastoma and pancreatic cancer, respectively, at a dose rate of 20 mg/kg/day for 14 days [19]. Based on this report, we administered 15 mg/kg/day of PKRA7 to CIA mice for 14 days from Day 21 and found that PKRA7 significantly suppressed the severity of arthritis. Because PKRA7 can bind to both PKR1 and PKR2 [19], the observed effect was likely the result of inhibition of both of the PK2-PKR1 and PK2-PKR2 pathways, possibly via suppression of neutrophil activation in the former and macrophage infiltration in the latter. In rheumatoid arthritis, macrophages infiltrate the synovial membrane and produce IL-1, IL-6, TNF-α, and other inflammatory cytokines [21]. In our study, administration of PKRA7 reduced IL-1 and IL-6 expression significantly as well as TNF-α gene expression levels to a lesser extent. These results suggest that PKRA7 may suppress the severity of arthritis by inhibiting PK2-PKR2 signaling in macrophages.

We previously proposed the possible role of PK2 in angiogenesis in arthritis based on elevated VEGF mRNA levels in CIA mice [15]. Indeed, PKRA7 has been shown to suppress angiogenesis, leading to tumor shrinkage in nude mice implanted with glioblastoma cells [19]. Unexpectedly, however, PKRA7 did not affect VEGF gene expression in our study. More study is necessary in order to understand the direct role of PK2 in angiogenesis. Since PK2 is a multifunctional protein that is likely involved in various processes of arthritis, a better understanding of these functions would be promising for the development of successful therapies for arthritis.

Our study has several limitations. First, we did not address the mechanism by which PKRA7 suppressed the severity of arthritis. Potential consequences of the inhibition of PK2-PKR1/2 signaling include alterations in immunomodulatory, inflammatory, and angiogenetic processes. Secondly, PK2, PKR1, and PKR2 expression has yet to be evaluated in human rheumatoid arthritis patients. Lastly, administration of PKRA7 to human patients may cause various side effects affecting pain thresholds, mental status, and sleep, as PK2 has diverse physiological roles. All of these questions are left to be answered by future study.

## Conclusions

PKRA7 suppressed the severity of arthritis and inflammatory cytokines in mice with collagen-induced arthritis. These results are encouraging for a possible application of the PK2 inhibitor as a new approach in the treatment of arthritis.

## Abbreviations

CIA: Collagen induced arthritis
PK2: Prokineticin 2
PKR1: Prokineticin receptor 1
PKR2: Prokineticin receptor 2

## References

[1] Negri L, Lattanzi R, Giannini E, Melchiorri P (2007). Bv8/Prokineticin proteins and their receptors. Life Sci.

[2] Martucci C, Franchi S, Giannini E, Tian H, Melchiorri P, Negri L, Sacerdote P (2006). Bv8, the amphibian homologue of the mammalian prokineticins, induces a proinflammatory phenotype of mouse macrophages. Br J Pharmacol.

[3] Franchi S, Giannini E, Lattuada D, Lattanzi R, Tian H, Melchiorri P, Negri L, Panerai AE, Sacerdot P (2008). The prokineticin receptor agonist Bv8 decreases IL-10 and IL-4 production in mice splenocytes by activating prokineticin receptor-1. BMC Immunol.

[4] Soga T, Matsumoto SI, Oda T, Saito T, Hiyama H, Takasaki J, Kamohara M, Ohishi T, Matsushime H, Furuichi K (2002). Molecular cloning and characterization of prokineticin receptors. Biochim Biophys Acta.

[5] Zhong C, Qu X, Tan M, Meng YG, Ferrara N (2009). Characterization and Regulation of Bv8 in Human Blood Cells. Clin Cancer Res.

[6] Shojaei F, Wu X, Zhong C (2007). Bv8 regulates myeloidcell-dependent tumour angiogenesis. Nature.

[7] Monnier J, Samson M (2010). Prokineticins in angiogenesis and cancer. Cancer Lett.

[8] De Felice M, Melchiorri P, Ossipov MH, Vanderah TW, Porreca F, Negri L (2012). Mechanisms of Bv8-induced biphasic hyperalgesia: increased excitatory transmitter release and expression. Neurosci Lett.

[9] Cheng MY, Bullock CM, Li C (2002). Prokineticin 2 transmits the behavioural circadian rhythm of the suprachiasmatic nucleus. Nature.

[10] Duncan WC, Nio-Kobayashi J (2013). Targeting angiogenesis in the pathological ovary. Reprod Fertil Dev.

[11] Urayama K, Guilini C, Messaddeg N, Hu K, Steenman M, Kurose H, Ert G, Nebigil CG (2007). The prokineticin receptor-1 (GPR73) promotes cardiomyocyte survival and angiogenesis. FASEB J.

[12] Matsumoto S, Yamazaki C, Masumoto KH, Nagano M, Naito M, Soga T, Hiyama H, Matsumoto M, Takasaki J, Kamohara M, Matsuo A, Kobori M, Katoh M, Matsushime H, Furuichi K, Shigeyoshi Y (2006). Abnormal development of the olfactory bulb and reproductive system in mice lacking prokineticin receptor PKR2. Proc Natl Acad Sci U S A.

[13] Kurosaka D, Hirai K, Nishioka M, Miyamoto Y, Yoshida K, Takahashi E, Ukichi T, Noda K, Yanagimachi M, Furuya K, Fukuda K, Yamada A (2009). Correlation between synovial blood flow signals and serum vascular endothelial growth factor levels in patients with refractory rheumatoid arthritis. Mod Rheumatol.

[14] Kurosaka D, Hirai K, Nishioka M, Miyamoto Y, Yoshida K, Noda K, Ukichi T, Yanagimachi M, Furuya K, Takahashi E, Kingetsu I, Fukuda K, Yamada A (2010). Clinical significance of serum levels of vascular endothelial growth factor, angiopoietin-1, and angiopoietin-2 in patients with rheumatoid arthritis. J Rheumatol.

[15] Kurosaka D, Noda K, Yoshida K, Furuya K, Ukichi T, Takahashi E, Yanagimachi M, Kingetsu I, Saito S, Yamada A (2009). Elevation of Bombina variegata peptide 8 in mice with collagen-induced arthritis. BMC Musculoskelet Disord.

[16] Chen B, Yu L, Wang J, Li C, Zhao K, Zhang H (2016). Involvement of Prokineticin 2 and Prokineticn Receptor 1 in Lipopolysaccharide-Induced Testis in Rats. Inflammation.

[17] Watson RP, Lilleyv E, Panesar M, Bhalay G, Langridge S, Tian SS, McClenagham C, Ropenga A, Zeng F, Nash MS (2012). Increased prokineticin 2 expression in gut inflammation: role in visceral pain and intestinal ion transport. Neurogastroenterol Motil.

[18] Abou-Hamdan M, Costanza M, Fontana E, Di Dario M, Musio S, Congiu C, Onnis V, Lattanzi R, Radaelli M, Salvadori S, Negri L, Poliani PL, Farina C, Balboni G, Steinman L, Pedotti L (2015). Critical role for prokineticin 2 in CNS autoimmunity. Neurol Neuroimmunol Neuroinflamm.

[19] Curtis VF, Wang H, Yang P, McLendon RE, Li X, Zhou QY, Wang XF (2013). A PK2/Bv8/PROK2 Antagonist Suppresses Tumorigenic Processes by Inhibiting Angiogenesis in Glioma and Blocking Myeloid Cell Infiltration in Pancreatic Cancer. PLoS One.

[20] Wright HL, Moots RJ, Edwards SW (2014). The multifactorial role of neutrophils in rheumatoid arthritis. Nat Rev Rheumatol.

[21] Mclnnes IB, Schett G (2011). The Pathogenesis of Rheumatoid Arthritis. N Engl J Med.

